# EMDataBank unified data resource for 3DEM

**DOI:** 10.1093/nar/gkv1126

**Published:** 2015-11-17

**Authors:** Catherine L. Lawson, Ardan Patwardhan, Matthew L. Baker, Corey Hryc, Eduardo Sanz Garcia, Brian P. Hudson, Ingvar Lagerstedt, Steven J. Ludtke, Grigore Pintilie, Raul Sala, John D. Westbrook, Helen M. Berman, Gerard J. Kleywegt, Wah Chiu

**Affiliations:** 1 Department of Chemistry and Chemical Biology and Research Collaboratory for Structural Bioinformatics, Rutgers, The State University of New Jersey, 610 Taylor Road Piscataway, NJ 08854, USA; 2 Protein Data Bank in Europe, European Molecular Biology Laboratory, European Bioinformatics Institute, Wellcome Genome Campus, Hinxton, Cambridge CB10 1SD, UK; 3 Verna and Marrs McLean Department of Biochemistry & Molecular Biology, National Center for Macromolecular Imaging, Baylor College of Medicine, 1 Baylor Plaza, Houston, TX 70030, USA

## Abstract

Three-dimensional Electron Microscopy (3DEM) has become a key experimental method in structural biology for a broad spectrum of biological specimens from molecules to cells. The EMDataBank project provides a unified portal for deposition, retrieval and analysis of 3DEM density maps, atomic models and associated metadata (emdatabank.org). We provide here an overview of the rapidly growing 3DEM structural data archives, which include maps in EM Data Bank and map-derived models in the Protein Data Bank. In addition, we describe progress and approaches toward development of validation protocols and methods, working with the scientific community, in order to create a validation pipeline for 3DEM data.

## INTRODUCTION

Three-dimensional electron microscopy (3DEM) is a powerful method for determining the 3D structures of large biological assemblies in solution and in the cell (1). Recent technological advances in cryo-electron microscopes, detectors and software have lead to a ‘quantum leap’ in 3DEM's capabilities, enabling elucidation of many previously inaccessible macromolecular complexes and subcellular architectures that carry out key biological processes (2,3). More and more frequently, near-atomic resolution is being achieved (4–6).

Density maps produced by 3DEM methods may be further interpreted through map segmentation, rigid body fitting of atomic coordinates determined using X-ray crystallography or NMR, and/or *ab initio* model building, depending on map resolution (7–9). EMDataBank, established in 2007 with funding from the National Institutes of Health/NIGMS as a joint effort of the National Center for Macromolecular Imaging, the Research Collaboratory for Structural Bioinformatics (RCSB) and the Protein Data Bank in Europe (PDBe), has unified data deposition and public access to maps and fitted models in EM Data Bank (EMDB) and Protein Data Bank (PDB), respectively (10,11). In our current funding period our major focus is on development of validation standards for 3DEM. The project website, EMDataBank.org, maintains access links to all project-related services (Table 1). We provide here an overview of the current state of the 3DEM structural data archives, progress toward development of a validation pipeline for 3DEM and future prospects.

**Table 1.** Overview of the EMDataBank.org site as of September 2015.

EMDataBank Site Page^a^	Description
deposit	Links to joint deposition servers hosted by RCSB PDB and PDBe, golden rules for deposition of EM data, EM map + model deposition guide, and EMDB hold/release policy, map format description, and FSC server
search	Links to simple search at RCSB and advanced EMDB search at PDBe
recententries	Table of all recently released and recently submitted EMDB entries, updated weekly
statistics	Links to EMDB distribution, trend, download, deposition & annotation statistics
allnews	EMDataBank.org news items
faq	Frequently asked questions about EM map volume depositions, EM fitted coordinate model depositions and map access
emdbaccess	Links to ftp archive sites, full archive download guide, EMDB web service
emsoftware	List of software available within the EM community for generation, analysis, and fitting of EM maps, sortable by program name and type
emtestdata	Links to EM image datasets and model challenge data that have been made publicly available by EM community members to download for testing
workshops	List of workshops that have been hosted by EMDataBank
emdbac	EMDataBank Advisory Committee meeting agendas, rosters, presentations and final reports

^a^Page links follow the form http://emdatabank.org/[pagename].html.

## EM STRUCTURAL DATA ARCHIVES

### EM archives overview

Public access to 3DEM maps and models is via the ftp archive of the Worldwide PDB (wwPDB (12)), which has distribution sites in USA, UK and Japan. The EMDB and PDB are housed in parallel branches, with both archives updated weekly on Wednesday at 0:00 UTC at all sites. EMDB holds 3DEM experimental data (density maps); PDB holds model coordinates derived from all major structural biology methods, including 3DEM. Correspondences between maps and associated fitted coordinate models are maintained within both archives. The underlying dictionaries for the two archives have mappings to one another and are regularly updated to reflect changes in experimental apparatus and methods.

**Figure 1.**
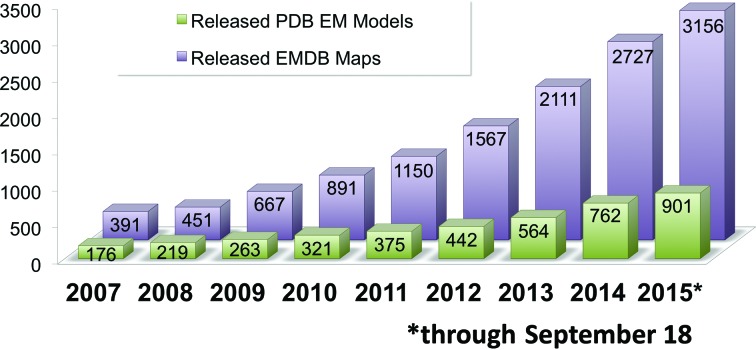
Released 3DEM entries in EMDB and PDB, cumulative by year, since the start of the EMDataBank Project in 2007.

The number of available EM structures in EMDB and PDB continues to grow robustly (Figure 1). As of September 2015, over 3100 maps and 900 models are available, with approximately 600 maps and 200 models being released per year. The annual growth rate is approximately 4-fold higher now than it was in 2010 (10). The observed growth reflects the increasing popularity of 3DEM methods to investigate large macromolecular complexes such as ribosomes, viruses and other macromolecular machines involved in protein folding, protein degradation, energy metabolism, cell cycle processes, DNA replication, DNA repair, RNA transcription, RNA splicing and ion transport.

**Figure 2.**
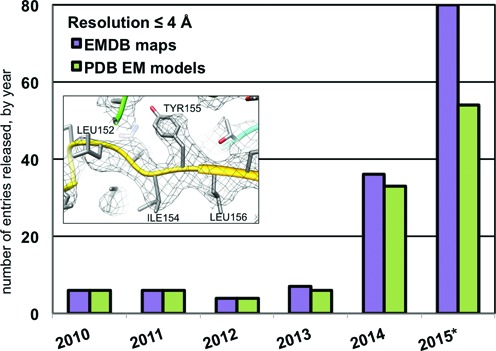
3DEM maps and models released each year between 2010 and 2015 (noncumulative) with reported resolution of 4 Å or better (*2015 data are through September 18). Inset: Segment of fitted density from the 3.8 Å map of Brome Mosaic Virus (30).

One of the most exciting recent trends in the 3DEM archives is a dramatic increase in maps produced, in which protein side-chains and nucleic acid bases can be resolved and models can be built directly into the map (Figure 2). This trend stems from the arrival of a new generation of direct electron detectors with much higher sensitivity, and image-processing tools that correct for sample movements and classify images according to different structural states (13–15). Although the vast majority of these new structures are in the 3.0–4.0 Å resolution range, recent landmark studies have reported single particle structures at 2.8 (16) and 2.2 Å (17), as well as an electron diffraction-derived structure at 1.4 Å (18). These results demonstrate the potential of 3DEM to deliver structures with unprecedented accuracy, and place its methods firmly at the cutting edge of structural biology research.

### EMDB content

Each EMDB entry describes a 3DEM imaging experiment and comprises a primary map, representative image and associated experimental metadata. Experimental metadata include information about the sample, specimen preparation, imaging, image processing, symmetry, reconstruction method, resolution and resolution method, as well as a description of the modeling/fitting procedures used. An EMDB entry may also include additional files related to the experiment, such as masks, structure factors derived from electron diffraction measurements, layer lines derived from helical images and Fourier Shell Correlation (FSC) curves used for map resolution estimation.

Since a single 3DEM imaging experiment can yield multiple density maps, additional maps may either be included within a single EMDB entry or divided among multiple entries, at the discretion of the depositing scientist. There are currently 84 entries with additional maps. The majority hold independently generated ‘half maps’ used for FSC calculation (e.g. 80S ribosome, EMD-2660 (19)). Other entries with additional maps hold segmented regions of the primary map (e.g. V-ATPase, EMD-1888 (20)), alternate conformations within a trajectory (e.g. 80S ribosome, EMD-6044 (21)) or additional representative tomograms (e.g. supercoiled DNA, EMD-6462 (22)).

**Figure 3.**
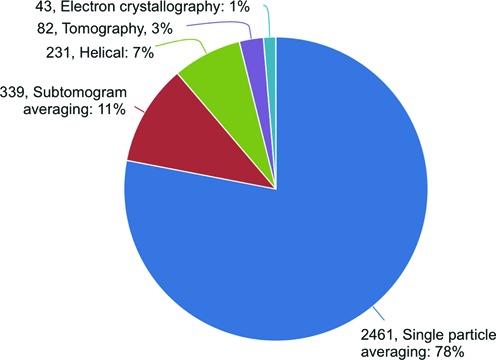
Distribution of released maps in EMDB (3156 total) as a function of 3DEM method used.

The current distribution of 3DEM methods represented in EMDB is shown in Figure 3. Single particle averaging, which produces maps that are ensemble averages of thousands of 2D images of individual particles, often with additional symmetry averaging (23), remains the most popular method, representing 78% of entries in the map archive. Subtomogram averaging, which produces maps that are also ensemble averages, but based instead on averaging 3D tomograms of individual particles (24), was not even recognized as a distinct method in EMDB 5 years ago (10), but is now the second most popular method (11%). Helical averaging (25), electron diffraction (26) and tomography (27) continue to be represented at small percentages, but all are actively developed with numbers of entries at least doubled since 2010.

### PDB EM content

3DEM PDB coordinate models are classified either under electron microscopy or electron crystallography as the experimental method. Each PDB entry comprises a fitted coordinate model and associated experimental metadata, fitting and refinement information, and primary sequence information for each polymer. For structures with regular point or helical symmetry, application of included transformation matrices will generate the full biological assembly from the provided asymmetric unit (28). Electron crystallography entries also include structure factors.

The percentage of PDB entries that are derived from 3DEM methods is still relatively small (<1% of ∼112 000 total entries), but growth has been significantly more rapid than for any other experimental method. In 2014, the number of structures from X-ray crystallography increased by 10% and the number of NMR structures increased by 5%, while the number of 3DEM structures increased by 36%. Of the 901 3DEM coordinate models that are now available, nearly half have been deposited in the past 3 years.

EM-derived coordinates are obtained using a variety of modeling methods including manual docking, rigid body fitting, homology modeling, *de novo* modeling and computational optimization algorithms. Tools originally developed for macromolecular model building and refinement against crystallographic data are now being adapted for cryoEM (29–31).

Structures in the PDB archive, and particularly those from 3DEM studies, are increasingly large and complex (32); those containing more than 62 polymer chains and/or more than 99 999 atoms cannot fit into a single PDB format file and have historically been ‘split’ across multiple entries. In late 2014 all structures that had previously been split were merged and integrated into the PDB archive as mmCIF-only entries. Fully one-third of PDB's 336 ‘large structure’ entries are 3DEM derived. The vast majority of these large structures are ribosomes, but viruses, membrane receptors, enzymes and designed DNA constructs are also represented.

### Deposition systems

Currently, joint EM map and model deposition systems are available at PDBe and RCSB (http://emdatabank.org/deposit.html). PDBe, RCSB and PDBj share 3DEM map and model annotation processing. Maps are first deposited to EMDB using a depositor-driven web-based deposition system, EMDEP (33), and are converted to a common format (based on the CCP4 crystallographic map format) for redistribution. Captured metadata including sample description, specimen preparation, imaging, reconstruction and fitting details are stored in an xml-style ‘header’ file that is distributed with the map. Additional map, mask and FSC curve files may also be uploaded (a server to prepare FSC files is described below). If a depositor wishes to deposit coordinates for a model fitted to the map, the experimental metadata collected in EMDEP are passed on to model deposition systems, either EM-ADIT (RCSB) or AUTODEP (PDBe), for subsequent submission to PDB, with correspondences between maps and associated fitted models maintained within both archives. Upon depositor request, EM maps and models may be held for up to 1 year from their respective deposition dates to EMDB and PDB. However, depositors are encouraged to make their data publicly available as soon as possible.

Shortly, joint deposition of 3DEM structures and associated experimental metadata to EMDB and PDB will become available through the wwPDB Deposition and Annotation (D&A) Tool, which has been available for X-ray structures since early 2014. The EMDataBank team has been working with the wwPDB Partners to develop and test the next version of this tool, which will be used at all wwPDB sites to curate structural data produced by any combination of experimental techniques (X-ray, NMR and 3DEM). Depositors will be able to complete map-only (EMDB) and combined map + model (EMDB + PDB) submissions, providing information tailored to the particular 3DEM method selected (single particle, helical, subtomogram average, tomography or electron crystallography). The new system will produce an EM-specific validation report (described below) and will feature a revised and expanded metadata dictionary for 3DEM experiments (34). For example, hierarchical sample description will be implemented in way that can be tied to map segmentations (35), and extensions for each 3DEM method will be added, following community-based recommendations (35,36).

### Search and visualization

Two search services are currently available through EMDataBank. EMSEARCH (10), hosted at RCSB, facilitates simple searches of EMDB based on author name, title, entry ID, sample name, citation abstract content, aggregation type, resolution and/or release date. Advanced EMDB search (37), hosted at PDBe, provides additional capabilities such as searches by sample molecular weight, taxonomy, reconstruction software package, microscope model and parameters, and has the ability to filter and further refine initial search results. Both search sites provide results listings with links to individual entry pages, where one can view overview information and access links for visualization tools and file downloads. The EMDB archive can also be investigated using the EMStats statistics service (37).

Three types of web-based visualization are available for 3DEM structures. First, a Java-applet-based volume viewer permits 3D visualization of maps and their associated PDB coordinate models (10,38). Second, a slice viewer is available for inspecting 2D slices of tomographic reconstructions (38). Third, visual analysis pages (34,37,38) facilitate analysis and validation of maps, tomograms and models by providing static images of map orthogonal projections and central slices as well as graphs of FSC curves, map density distribution, rotationally averaged power spectrum, volume estimation versus contour level and model atom inclusion at the recommended contour level. These visualization tools have been designed to help nonexperts and experts alike to gain insight into the content and assess the quality of 3DEM structures in EMDB and PDB without the need to install specialized software or to download large amounts of data from the structural data archives.

Maps and associated model files may also be downloaded for local analysis via links on individual entry pages. EMDB maps can be viewed along with associated models using locally installed software such as UCSF Chimera (39), Pymol (40), VMD (41) and Coot (42), enabling investigation with an extensive set of tools.

## EM VALIDATION DEVELOPMENT

Assessment of structural data crucially requires community-accepted validation criteria (36,43,44). However, methods for validation of 3DEM structures are still in early development, and are applied inconsistently (36,45–48). Engaging the 3DEM community, the EMDataBank project team is working to establish data validation methods that can be used in the structure determination process, to define key indicators of a well-determined structure that should accompany every structure deposition and to implement appropriate validation procedures into a 3DEM validation pipeline.

In 2010, we established the EM Validation Task Force (EM VTF), which strongly articulated the need for more research and development of validation criteria for maps and map-derived models (36). The EM VTF also recommended providing full FSC curves with deposited maps, indicating whether or not maps used for FSC calculation are fully independent, establishing benchmark datasets for maps and models and providing multiple types of assessments for models. Participants at the 2011 Data Management Challenges in 3D Electron Microscopy (DMCEM) workshop reiterated the EM VTF's advice, and provided further recommendations regarding development of data models and validation-related services (34).

We are following up on these recommendations with research efforts, community-wide challenges and validation pipeline development. Ongoing research activities include development and testing of protocols to define best practices in model optimization using modified crystallography modeling packages, cross-validation of *de novo* models using independently determined half-maps (30,31) and development of new validation strategies for maps (49). Challenge and pipeline development progress is described below.

## Map and model challenges

**Figure 4.**
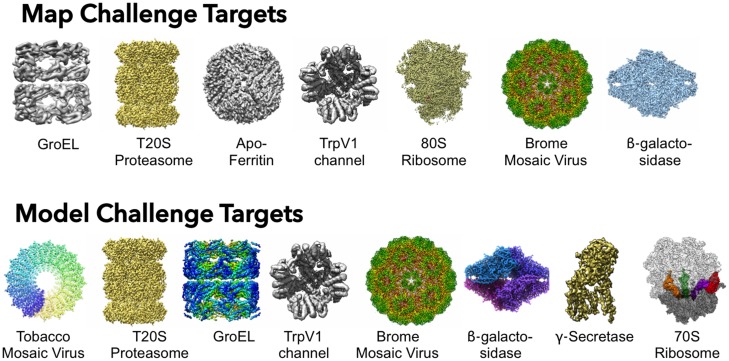
Target structures for the 2015 Map and Model Challenges, chosen by the respective 3DEM-community based committees.

EMDataBank is sponsoring two new community challenges in 2015–2016 to raise awareness of the need for structure validation as a routine part of 3DEM studies and publications. Additional goals are to develop suitable sets of benchmark data, establish best practices, evolve criteria for validation and compare and contrast different 3DEM methodologies. The new challenges have been formulated by committees composed of 3DEM community members, with benchmark targets of varying size and complexity selected from recently deposited 3DEM structures based on current state-of-the-art detectors and processing methods, in the resolution range 2.2–4.5 Å (Figure 4). The new challenges follow in the positive spirit of previous community-based challenge activities for particle picking (50), modeling (51) and CTF correction (52). We anticipate that the community-developed benchmarks will prove useful for methods evaluation, even beyond these challenge activities (53).

For the map challenge, participants are asked to create and submit reconstructions using supplied image data. The map challenge data, which totals 12 TB for seven benchmark targets and includes raw movie frame images, have been provided by the original authors of the selected targets, and are stored in a new pilot archive for raw 2D image data being developed by PDBe (EMPIAR, http://pdbe.org/empiar).

For the model challenge, participants are asked to create and submit atomic coordinate models using supplied maps. Following a key recommendation of cryoEM specialists and modeling software developers at a planning workshop we organized in June 2015, each benchmark target is an unfiltered, unsharpened map. Half-maps used for FSC curve calculation are also available for participants to try out various refinement and validation strategies. Maps for the eight targets have been provided by the original authors of the target structures, and are stored as supplemental files associated with EMDB entries.

Each challenge will have challenger submission and results assessment phases. Follow-up discussions via participant workshops are planned, as well as dissemination of results in journal special issues. Anyone from the scientific community is welcome to participate as a challenger and/or assessor. Both challenges are hosted at http://challenges.emdatabank.org.

### Validation services

Two validation servers have been developed at PDBe for eventual integration into the 3DEM validation pipeline.

**Figure 5.**
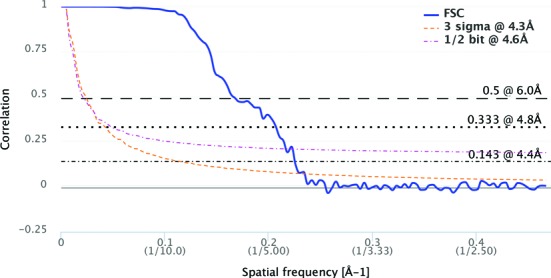
Example Fourier–Shell Correlation curve plot produced by the FSC server. Estimated resolution values according to a variety of commonly used criteria are provided at the right.

FSC (54) is the most commonly reported method for estimating the resolution of single-particle maps. However, the estimated resolution depends critically on the threshold criteria used, and several different conventions are followed. In order to simplify deposition of FSC curve data to EMDB, a web service for calculating FSC curves has been developed, community-tested and placed into production (http://pdbe.org/fsc). A user can upload two independent maps, receive the calculated FSC curve in a standardized format for deposition into EMDB, and view and download a plot of the curve (Figure 5). The server uses the EMAN2 (55) package for FSC calculation. Several reconstruction packages also produce FSC files suitable for direct upload to EMDB including EMAN2, RELION (56) and Bsoft (57). More than 120 map entries in EMDB now include deposited FSC curves.

Tilt-pair analysis (58,59) is a useful method for validating the hand and overall shape of a map, particularly for lower resolution maps in which secondary structure features are absent. A tilt-pair validation server developed by the Rosenthal group (60) has made the method generally accessible. This server has now been migrated to PDBe and is available for public use (http://pdbe.org/tiltpair).

### Validation report

**Figure 6.**
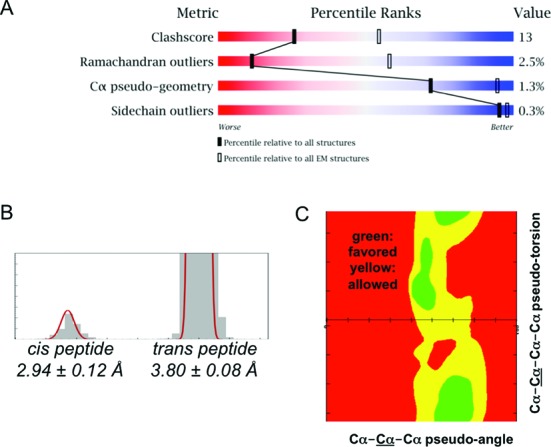
EM validation report elements. (**A**) Slider graphic, which provides an impression of the overall quality of an EM model at a glance. (**B**) Bimodal distribution of protein Cα–Cα distances. (**C**) Distribution of protein Cα–pseudo angles and pseudo torsion angles. The distributions shown in (B) and (C) are derived from high-resolution X-ray structures and are used in the report to identify trace atom model geometry outliers.

An initial EM validation report has been developed for use in the D&A system (Figure 6). The format closely follows the validation reports produced by wwPDB for structures from X-ray crystallography (43) and NMR (44), and is based on the same underlying validation software pipeline (61). We have initially focused on providing map-independent assessments of model quality. Model assessments include standard geometry (bonds, angles and torsion angles), close contacts, protein and nucleic acid backbone geometry, and ligand geometry. A slider graphic compares the quality of the given structure, for key indicators, to all EM structures in the PDB archive, as well as all structures in the PDB archive (Figure 6A).

Recognizing that nearly one quarter of all 3DEM models in the PDB contain polymers represented as atom traces (Cα-atom only for protein chains; P-atom only for nucleic acid chains), we have developed new assessments for trace atom model geometry (62). Consecutive Cα–Cα distances are reported as outliers if they fall outside ±3σ limits for *cis* and *trans* peptide distributions (Figure 6B); consecutive P–P distances are reported as outliers if they are shorter than 4.4 Å or longer than 8.0 Å. Regions with poor Cα trace geometry are also reported based on pseudo-Ramachandran analysis (62) (Figure 6C).

The EM validation report also provides a table of basic information about the map, e.g. the reconstruction method, reported resolution, resolution method, imposed symmetry, number of images used, microscope, imaging parameters and detector. Future report versions will, with guidance from the EM VTF, add validation components for the map as well as for the fit of the model to the map, as the relevant methods and metrics evolve and become accepted community standards. We will encourage depositors to include the validation report when submitting manuscripts for review, and encourage journal editors and reviewers to request these reports.

## FUTURE PROSPECTS

3DEM structures are giving us an unprecedented understanding of biology, but, as was the case for X-ray crystal structures three decades ago, the ability to assess the quality of 3DEM structures is limited. The efforts by the X-ray crystallographic community to establish standards, protocols and methods for validation of experimental data have made these structures enormously valuable for understanding function and developing structure prediction methods.

We aim through engagement with the 3DEM community to create for the first time a consensus set of validation procedures and statistical assessments for 3DEM data, maps and models. Validation results will either be included as data items upon deposition (e.g. statistics pertaining to the quality of the raw data) or will become part of the validation pipeline for the deposited maps and models. This will enable both producers and users of 3DEM data to evaluate quality and reliability and the extent to which the experimental data support structural, functional and mechanistic inferences, hypotheses and interpretations.
